# A Meta-Analysis of Obesity and Risk of Colorectal Cancer in Patients with Lynch Syndrome: The Impact of Sex and Genetics

**DOI:** 10.3390/nu13051736

**Published:** 2021-05-20

**Authors:** Matteo Lazzeroni, Federica Bellerba, Mariarosaria Calvello, Finlay Macrae, Aung Ko Win, Mark Jenkins, Davide Serrano, Monica Marabelli, Sara Cagnacci, Gianluca Tolva, Debora Macis, Sara Raimondi, Luca Mazzarella, Susanna Chiocca, Saverio Caini, Lucio Bertario, Bernardo Bonanni, Sara Gandini

**Affiliations:** 1 Division of Cancer Prevention and Genetics, European Institute of Oncology (IEO) IRCCS, 20141 Milan, Italy; mariarosaria.calvello@ieo.it (M.C.); davide.serrano@ieo.it (D.S.); monica.marabelli@ieo.it (M.M.); sara.cagnacci@ieo.it (S.C.); debora.macis@ieo.it (D.M.); lucio.bertario@gmail.com (L.B.); bernardo.bonanni@ieo.it (B.B.); 2 Department of Experimental Oncology, European Institute of Oncology (IEO) IRCCS, 20141 Milan, Italy; Federica.Bellerba@ieo.it (F.B.); sara.raimondi@ieo.it (S.R.); luca.mazzarella@ieo.it (L.M.); susanna.chiocca@ieo.it (S.C.); sara.gandini@ieo.it (S.G.); 3 Department of Colorectal Medicine and Genetics, Royal Melbourne Hospital, Parkville, VIC 3050, Australia; finlay.macrae@mh.org.au; 4 Centre for Epidemiology and Biostatistics, University of Melbourne, Parkville, VIC 3050, Australia; awin@unimelb.edu.au (A.K.W.); m.jenkins@unimelb.edu.au (M.J.); 5 Victorian Comprehensive Cancer Centre, University of Melbourne Centre for Cancer Research, Parkville, VIC 3050, Australia; 6 Cancer Risk Factors and Lifestyle Epidemiology Unit, Institute for Cancer Research, Prevention and Clinical Network (ISPRO), 50139 Florence, Italy; s.caini@ispo.toscana.it

**Keywords:** lynch syndrome, colorectal cancer, gender difference, body weight

## Abstract

There appears to be a sex-specific association between obesity and colorectal neoplasia in patients with Lynch Syndrome (LS). We meta-analyzed studies reporting on obesity and colorectal cancer (CRC) risk in LS patients to test whether obese subjects were at increased risk of cancer compared to those of normal weight. We explored also a possible sex-specific relationship between adiposity and CRC risk among patients with LS. The summary relative risk (SRR) and 95% confidence intervals (CI) were calculated through random effect models. We investigated the causes of between-study heterogeneity and assessed the presence of publication bias. We were able to retrieve suitable data from four independent studies. We found a twofold risk of CRC in obese men compared to nonobese men (SRR = 2.09; 95%CI: 1.23–3.55, I^2^ = 33%), and no indication of publication bias (*p* = 0.13). No significantly increased risk due to obesity was found for women. A 49% increased CRC risk for obesity was found for subjects with an *MLH1* mutation (SRR = 1.49; 95%CI: 1.11–1.99, I^2^ = 0%). These results confirm the different effects of sex on obesity and CRC risk and also support the public measures to reduce overweight in people with LS, particularly for men.

## 1. Introduction

Colorectal cancer (CRC) accounts for approximately 10% of all annually diagnosed cancers and cancer-related deaths worldwide [1]. Of all new cases of CRC, 3% are attributable to hereditary nonpolyposis colorectal cancer (HNPCC) or Lynch syndrome (LS) [2]. LS runs in families in an autosomal dominant inheritance pattern and is the most common cause of hereditary colorectal cancer [3]. The diagnosis is made upon identification of a germline mutation in a mismatch repair (MMR) gene (*MLH1*, *MSH2*, *MSH6*, or *PMS2*) or a germline deletion in an epithelial cell adhesion molecule (*EPCAM*), which leads to epigenetic inactivation of *MSH2* [4,5]. Depending on the affected gene, people with LS have a lifetime risk of CRC up to 50% and a younger age of onset [6]. Due to the high penetrance of this condition, people with LS may be advised to take a daily aspirin to reduce their risk of CRC, according to the recent draft guidance of the National Institute for Health and Care Excellence (NICE) [7]. The biological basis of this recommendation lies in the concept that cancer development in LS can be modulated by environmental factors, particularly those that influence inflammation [8]. In the general population, there is considerable evidence that adults with higher obesity are at higher risk for several common cancers, including CRC [9]. However, it remains unclear whether the effect of adiposity on CRC risk differs among men and women [10,11,12]. In principle, overweight or obese LS patients may be at an even higher risk of cancer than normal-weight LS patients, because of the reduced ability to repair DNA damage. In practice, the picture is considerably less clear. To clarify whether sex and MMR genes could be modifiers of risk, while also considering the ongoing obesity epidemic and the difficulties in reducing adiposity itself [13,14], we performed a systematic literature review and meta-analysis of studies reporting the association between obesity and CRC risk in patients with LS.

## 2. Materials and Methods

This literature review and meta-analysis was designed, conducted, and described according to the MOOSE guidelines for systematic reviews and meta-analyses of observational studies [15]; the study protocol was submitted in the PROSPERO register of systematic literature reviews. Prospero registration number: CRD42020172075.

### 2.1. Eligibility Criteria and Study Selection

We carried out a meta-analysis based on published evidence to investigate whether sex and MMR genes may modify the effect played by obesity on the development of CRC in patients with LS. The primary inclusion criteria identified for potentially eligible studies for this meta-analysis were the following: (i) the studies should include subjects with LS or subjects who met the Amsterdam/Bethesda (revised) criteria for LS [16]; CRC risk was evaluated as study endpoint (if there was not a risk estimate for CRC we included the estimate for colorectal adenoma as a proxy) [17]; (ii) the manuscript includes risk estimates and 95% confidence interval (CI) for BMI/weight status assessment; (iii) the studies have to be independent; the study design is a cohort study or a case-control study. The study presented the risk estimates stratified by sex. When more estimates were reported, we preferred estimates for the incident to prevalent cases and estimates for current BMI to BMI at age 20. We also retrieved CRC risk estimates for obesity by single MMR gene mutations, when available. A sensitivity analysis was carried out including also the risk estimate from the study that did not report data stratified by sex. 

### 2.2. Statistical Analysis

As CRC is a rare event in the population, all estimates of risk (odds ratio, hazard ratio) for obese versus non-obese subjects were considered a good approximation of relative risk (RR). Every measure of association and its 95% CI were log-transformed and the corresponding standard error was calculated by using the formula proposed by Greenland [18]. Summary RR (SRRs) by sex were estimated by pooling the log-transformed estimates provided by each study with a random effects model [19], to account for both within and between studies variations. A summary risk estimate was calculated when at least three estimates were available. The homogeneity across the studies was verified with a test based on Cochran’s Q statistic, which is distributed as a Chi-square with k-1 degrees of freedom, where k is the number of studies. The Higgins and Thompson’s I^2^ statistic, which ranges from 0% to 100%, was provided to quantify the percentage of total variation across studies that is attributable to heterogeneity rather than chance. A threshold of I^2^ below 50% was considered an acceptable level of between-study heterogeneity [20]. A possible source of bias and quality was assessed using the STROBE checklist and a modified version of the Newcastle−Ottawa scale [21]. A sensitivity analysis was carried out including all available estimates of the association (stratified and overall), to evaluate whether there was a significant overall effect, independent of sex. Publication bias was graphically represented with funnel plots and evaluated with the Macaskill test [22], which is based on the regression of the ln(HR) or ln(OR) on the sample size and weighted by the inverse of the pooled variance. All the statistical analyses were performed using SAS software version 9.4 (SAS Institute, Cary, NC, USA).

### 2.3. Search Strategy

According to the “Population-Item-Comparison-Outcome” (PICO) framework, the population of interest was exclusively composed of people with LS or subjects who met the Amsterdam/Bethesda (revised) criteria. The exposure of interest was the sex-specific relationship between adiposity and colorectal neoplasia. The literature review was conducted in Medline and EMBASE for papers published up to 31 December 2019, using the following search string: (“lynch” OR hereditary non-polyposis colon cancer” OR “HNPCC”) AND (“weight” OR “obesity” OR “BMI” OR “adipose” OR “adiposity”) AND (“colorectal” OR “colon” OR “rectal”) AND (“cancer” OR “tumor”). The controlled vocabulary and keywords included in the search string were designed to ensure that all published evidence regarding weight/BMI and CRS in subjects with LS would be covered. After removing duplicates entries, an initial screening based on title and abstract was made independently by three researchers (FB, SG, and ML), and papers were discarded when there was consensus among the panel members. Papers that were instead deemed potentially suitable by at least one researcher were read in full by all panel members, who independently verified that all inclusion criteria were met. Any disagreements were resolved by consensus. Bibliography of relevant studies was also checked to further improve the search and confirm that all information was recruited. No time or language restriction was applied. Three independent authors (FB, SG, and ML) who selected studies and extracted relevant data conducted a comprehensive literature search.

## 3. Results

**Figure 1:**
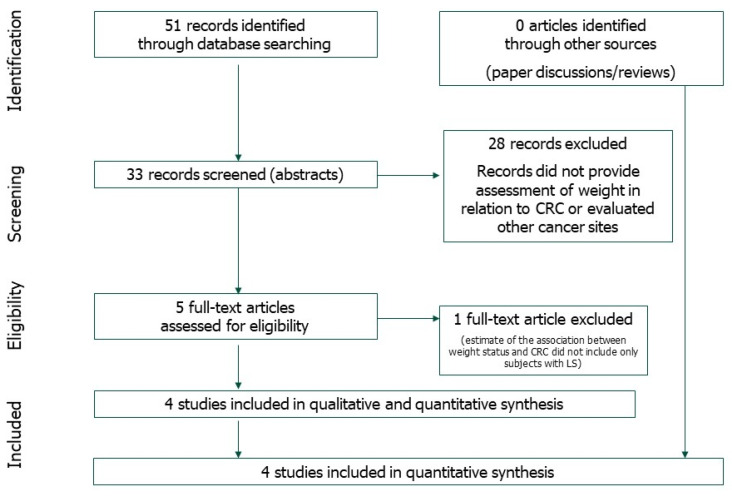
Selection process (flowchart).

The literature search produced 51 items. Eighteen were excluded by the consensus panel. depending on the title and the abstract of a research paper, (Figure 1). We read 33 articles in the full version. After removing 28 papers that did not provide an assessment of weight-related CRC or evaluated other cancer sites, a total of five studies published between 2007 and 2015 were included (Figure 1).

One case-control study [23] was not included in the meta-analysis because the estimate of the association between weight status and CRC did not include only subjects with LS. Studies included in the meta-analysis are summarized in Table 1. 

Three of the selected studies [24,25,26] are prospective studies and one [27] is a case-control study. Furthermore, one study [24] used data from the “Genetic, environmental and other influences among persons with LYNCH syndrome” (GEOLynch) Cohort, one [26] from the “Colorectal Adenoma/Carcinoma Prevention Programme (CaPP) 2 trial, one [25] from the Colon Cancer Family Registry. The number of patients varied from 265 to 3595. Three of the four studies provided estimates for the association between obesity and CRC stratified by sex, whereas the study by Win et al. [25] reported only the overall estimate. In this last case, we retrieved the risk estimates of CRC for men and women from the authors by personal communication. Obesity was identified as a BMI greater than 30 kg/m2, except Botma et al. [24], which presented risk estimates for overweight and obese subjects together (i.e., for BMI ≥ 25 kg/m^2^). For Win et al. [25] the estimates refer to BMI “at age 20”, while all the other studies considered “current” BMI. Three of the four studies provided estimates for the association between obesity and CRC stratified by *MLH1* and *MSH2* genes. All studies with a prospective study design expressed the association between obesity and CRC in terms of adjusted HR (95% CI), while the case-control study reported the adjusted OR (95% CI). 

**Figure 2:**
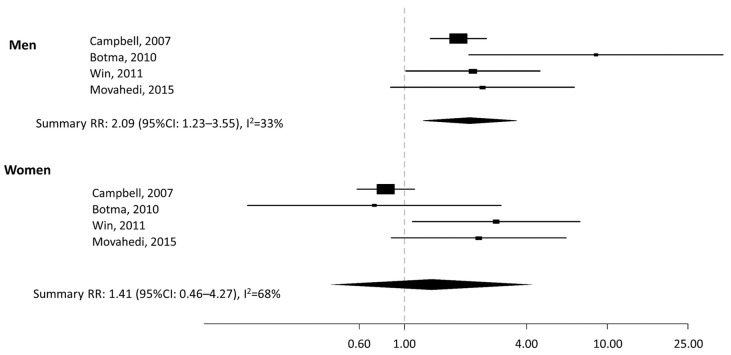
Forest plot of the association between colorectal cancer risk for obese vs. nonobese in subjects with Lynch Syndrome by sex. RR: relative risk. CI: confidence intervals.

All the studies assessed the risk for CRC, except for Botma et al. [24] who evaluated colorectal adenoma risk. As shown in the forest plot (Figure 2), the SSR indicates a twofold higher risk of CRC in obese men compared to nonobese men (SRR = 2.09; 95%CI: 1.23–3.55, with average between-study heterogeneity I^2^ = 33%), with no indication of publication bias (*p* = 0.13). No significant difference was found between obese versus nonobese women (SRR = 1.41, 95%CI: 0.46–4.27, with a between-study heterogeneity I^2^ = 68%).

Table 2 shows the relationship between BMI and CRC risk in LS patients by MMR gene.

**Figure 3:**
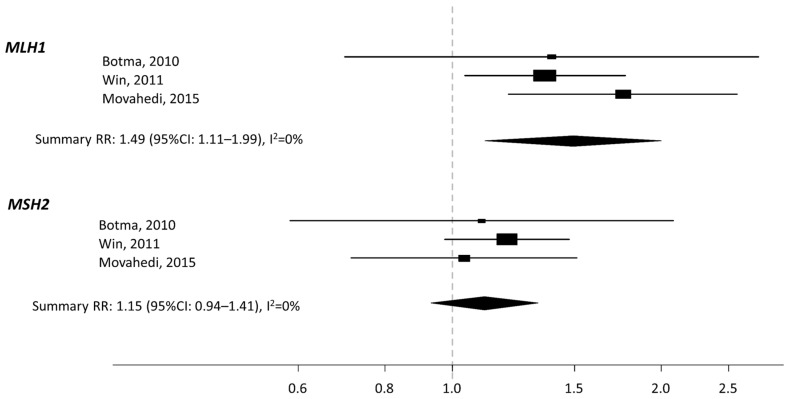
Forest plot of CRC risk estimate for *MLH1* and *MSH2*, for increasing value of BMI by 5 kg/m^2^.

We calculated a summary risk estimate for *MLH1* and *MSH2*, for increasing the value of BMI by 5 kg/m^2^ (Box-plots in Figure 3). 

In subjects with a mutation in *MLH1*, we found a significantly increased risk of 49% for every increase of 5 kg/m^2^ (summary RR 1.49; 95% CI: 1.11–1.99), with no indication of heterogeneity, I^2^ = 0%. No association was found for *MSH2*: summary RR: 1.15 (95%CI: 0.94–1.41) every increase of 5 kg/m^2^, with no indication of heterogeneity, I^2^ = 0%. The quality of the studies was generally very high (Supplementary Table S1). 

## 4. Discussion

LS is the most common hereditary cancer syndrome, affecting an estimated 1 in 370 individuals [28]. Pathogenic variants in each of the MMR genes result in different cancer risks for different organs, mainly colorectum and endometrium, but also including ovaries, stomach, small bowel, bile ducts, pancreas, and upper urinary tract. In Italy, no structured and standardized pathways for the diagnosis and management of LS patients are currently in place, apart from a few high-risk clinics [29]. Further to the very recent data demonstrating different cancer risks by gene, age, and sex, in carriers of the pathogenic MMR variants [30], we performed a systematic review and meta-analysis of observational studies on the association between obesity and CRC risk among LS patients. We found that obesity was associated with a significantly increased risk of CRC in men, but not in women. Our results give a broader and more comprehensive view of the risk of CRC in obese subjects with LS and complement the findings of previous studies on obesity and CRC risk in the general population, where the excess of body weight in men is associated with a significantly higher risk of CRC than excess body weight in women [31]. The hypothesis that, given the germline loss of MMR function in LS, the obesity-related chronic inflammation might have a promoting effect on those stem cells with acquired DNA damage due to this failing system of repair, should be applied to both sexes. However, beyond the documented existence of sex differences in obesity-induced inflammation in carcinogenesis [32], the additive effect of obesity might be compensated in women by the qualitative and quantitative effect of hormone exposure, including both reproductive behavior (number of pregnancies, age at first birth) and use of oral contraceptive and hormone replacement therapy. A plausible explanation of this lower CRC incidence in women may also lie in a tissue-specific difference where gastrointestinal tract tissue might differ from other tissues in its handling of exposure to hormones. The estrogen signaling mediated by ERβ has been shown to exert multiple antitumorigenic effects in the colonic mucosa, including the modulation of immune surveillance mechanisms, the inhibition of inflammatory signals, and the induction of apoptosis [33]. Lastly, we must also consider that more than 50% of women with LS will develop a gynecologic malignancy as their sentinel cancer [34], thereby influencing CRC incidence in women. 

Very recent findings from a prospective cohort of 6350 carriers of pathogenic mutations in MMR genes have now imposed a revision of the management guidelines, considering different gene and sex-specific risks [30]. The lifetime risk of CRC in pathogenic (path_) variants of *MLH1* and *MSH2* was approximately 50%, despite attempted prevention by surveillance colonoscopy and polypectomy [30]. The risk was higher in the male than in the female path_*MLH1* carriers, whereas in early adulthood, path_*MSH2* carriers of both sexes had the same high CRC risk. The low incidence of CRC in path_*MSH6* carriers (18% lifetime risk) appears to be a sex-limited trait with rather low penetrance in men [30]. Lastly, heterozygous carriers of path_*PMS2* variants had no increased risk of CRC, irrespective of sex (notably, CRC risk was not increased before age 50, with a nonsignificant increase at older ages) [30]. 

In addition to nonmodifiable factors, such as genes and sex, we found that adiposity may also significantly increase the risk of CRC in men with a genetic predisposition, thus better depicting the concept of “incomplete penetrance”. This is a term often criticized as reflecting the lack of knowledge of genetic and environmental factors that may interact with the genotype to determine the ultimate phenotype of the individual. It is even more intriguing to find that the increased risk due to adiposity in subjects with LS seems to be limited to path_*MLH1* carriers. Very recently, data suggested possible different pathways of CRC development in *MLH1* vs. *MSH2* carriers [35]. In particular, whereas *MSH2*-associated CRCs have a higher frequency of somatic *APC* mutations compared with *MLH1*-associated CRCs, a significantly higher frequency of *CTNNB1* mutations has been observed in *MLH1*-associated CRCs compared with *MSH2*-associated ones [35]. Obesity and inactivity are associated with an increased risk for *CTNNB1*-negative CRCs and with every 5 kg of body fat (measured by BMI), the risk for *CTNNB1*-negative colorectal cancer increases by about one-third [36]. All these data, taken together, may explain, at least in part, our findings. 

The guidelines committee of NICE published draft guidance advising physicians that people with LS should be offered daily aspirin to reduce their risk of CRC [7]. The CaPP3 double-blind noninferiority phase III trial is now looking at the effects of three different doses of aspirin (600 mg, 300 mg, 100 mg) [37]. Because participant BMIs are being documented, the results will probably answer both the questions of the optimal dose in the prevention setting and the obesity/sex/aspirin dose debate. Without the counterbalance of estrogen, men with LS could ideally rely upon a synergistic effect of aspirin and weight loss, given the intriguing positive effects that weight loss has demonstrated, for example, in obese patients undergoing gastric bypass surgery [38]. Following surgery and consequent weight loss, participants showed improvements in systemic markers of inflammation (e.g., CRP), a significant fall in the expression of the proinflammatory gene *COX-1*, and a reduced total mitosis in the crypt in the colorectal mucosa [38]. 

These results might be achievable by promoting weight loss by lifestyle changes (reduced dietary energy intake and increased energy expenditure in physical activity) [39]. A feasibility study has in fact shown that lifestyle-based interventions may be possible and acceptable in those with a family history of CRC, such as LS patients [40]. 

The main strengths of our meta-analysis are the fullness of the literature search, despite the number of the included studies on this specific topic, and the calculation of summary CRC risk estimates in both obese men and women with LS. Despite the good methodological quality of most included studies, there is a substantial variability in terms of study design, methods used to define exposure definition, and statistical methods, potentially affecting the study results. This resulted in a considerable between-estimates heterogeneity, thus highlighting the necessity to standardize methods to get comparable risk estimates and reach more robust conclusions. Another limitation of all the included studies was that height and weight (which were used to calculate BMI) were self-reported. Since it is well known that height and weight are often overestimated and underestimated, respectively, absolute rates of obesity are frequently underestimated [41]. Self-reported height and weight, on the other hand, have a high sensitivity and specificity for detecting obesity, 0.83 and 1.00, respectively [41]. Secondly, the study of Campbell [27] analyzed BMI in association with the risk of colorectal cancer, stratified by sex and family history of CRC status (Amsterdam/Bethesda criteria), not by germline mutation data. Furthermore, our meta-analysis did not allow us to study the effect of BMI on CRC risk in path_*PMS2* and path_*MSH6* carriers, where, precisely because of the low penetrance of these two genes, adiposity could have been a decisive modifier. Finally, Botma et al. [24] analyzed the BMI associated with the risk of colorectal adenomas and not CRCs, although it should be kept in mind the higher adenoma−carcinoma progression ratio of CRCs in LS, compared to sporadic cases [42].

Despite the abovementioned limitations, because the available evidence about the effects of weight loss on CRC risk is limited, especially in subjects with LS, along with the difficulty to perform large randomized clinical trials in patients with rare conditions such as LS, we think our meta-analysis takes on extra significance and supports future research. In the meantime, public measures to reduce the development of obesity and to enable those who are obese to lose weight (at least 20% of their body weight) [43] are mandatory, since such interventions are likely to benefit all sections of the population, including those at higher risk due to familial conditions such as LS.

## Figures and Tables

**Table 1:** Main characteristics of the studies included in the meta-analysis.

FA	PY	Study Names	Country	Study Design	Controls/Size Cohort	Cases/Events	Contrast	Inclusion Criteria
Campbell	2007		Canada	CC	2668	927	Obese vs. normal, current weight	Member of AC-I or RBG families
Botma *	2010	GEOLynch	The Netherlands	Cohort	243	22	Obese/overweight vs. normal, current weight	*MLH1*, *MSH2*, *MSH6*, *PMS2* carriers
Win	2011	CCFR	Australia, North America	Cohort	1324	659	Obese vs. normal, at age 20	*MLH1*, *MSH2*, *MSH6*, *PMS2* carriers
Movahedi	2015	CAPP2 trial	Australia, China, Europe, South Africa, USA	Cohort	896	54	Obese vs. normal, current weight	*MLH1*, *MSH2*, *MSH6* carriers or AC-I families

FA = first Author; PY = publication Year; CC = case-control; CCFR = Colon Cancer Family Registry; AC-I = Amsterdam criteria I; RBG = revised Bethesda guidelines; * risk of colorectal adenoma.

**Table 2:** Correlation between BMI and CRC risk in LS patients by MMR gene.

Author, PY	Data Source	Country	Study Design	Outcome	BMI Evaluation	Gene	HR (95%CI)
Botma et al. 2010	GEOLynch	Netherlands	Cohort study	Colorectal Adenoma	Per 5 kg/m^2^, current Overweight or obese vs. normal, current	*MLH1*	1.39 (0.70–2.76) *
						*MSH2*	1.14 (0.47–2.74) *
						*MSH6*	2.77 (0.19–40.27) *
						*MLH1*	2.64 (0.47–14.89) *
						*MSH2*	1.08 (0.21–5.73) *
						*MSH6*	4.69 (0.62–35.61) *
Movahedi et al. 2015	CAPP2 trial	Australia, China, Europe, South Africa, USA	Cohort study	CRC	Per 1 kg/m^2^, current Overweight vs. normal, current Obese vs. normal, current	*MLH1*	1.12 (1.04–1.21) ^+^
						*MSH2*	1.01 (0.91–1.12) ^+^
						*MLH1*	1.19 (0.47–3.01) ^+^
						*MSH2*	1.26 (0.44–3.60) ^+^
						*MLH1*	3.72 (1.41–9.81) ^+^
						*MSH2*	1.59 (0.47–5.44) ^+^
Win et al. 2011	CCFR	Australia, North America	Cohort study	CRC	Per 5 kg/m^2^, at age 20	*MLH1*	1.36 (1.04–1.77) ^#^
						*MSH2*	1.28 (0.96–1.70) ^#^
						*MSH6*	0.84 (0.38–1.80) ^#^

* Adjusted for age, smoking habits, and alcohol intake. All estimates refer to the incidence cohort. ^+^ Adjusted for age, sex, starch, aspirin, and geographic region. ^#^ Adjusted for sex, country, cigarette smoking and alcohol drinking with robust variance estimation for familial correlation in risk.

## Data Availability

Data used for the meta-analysis are available through the Senior Author (SG) upon request.

## Supplementary Materials

The following are available online at https://www.mdpi.com/article/10.3390/nu13051736/s1, Table S1. The Newcastle-Ottawa Scale (NOS) for Assessing the Quality of Nonrandomized Studies included in the Meta-Analysis.
