# Supplementary material for: A Meta-Analysis of Obesity and Risk of Colorectal Cancer in Patients with Lynch Syndrome: The Impact of Sex and Genetics
Source: Nutrients. 2021 May 20;13(5):1736. doi: 10.3390/nu13051736 (PMC8160758; doi:10.3390/nu13051736)
Supplement: Supplementary file 1 [file nutrients-13-01736-s001.zip › nutrients-1204737-supplementary.pdf]

Supplementary Table . Quality assessment of studies using a modified Newcastle-Ottawa scale

Table S1. Quality assessment of studies using a modified Newcastle-Ottawa scale-COHORT STUDIES

Quality assessment criteria	Accettable (*)	Botma et al.	Movahedi et al.	Win et al.
Selection				
Representativeness of exposed cohort?	Cancer registries/Amsterdam diagnostic criteria	*	*	*
Selection of the non-exposed cohort?	Same setting as exposed cohort	*	*	*
Ascertainment of exposure?	Measured BMI			
Demonstration that outcome of interest was not present at start of study?	Information about previously colonoscopies and adenomatous polyp occurrences	*	*	*
Comparability				
Stratification for possible modifier of risk	Study stratify for sex	*	*	
Study controls for at least 3 additional risk factors?	Adjusted for age, smoking habits, alcohol intake, aspirin and/or genes	*	*	*
Outcome				
Assessment of outcome?	Secure record or directly measured of colorectal cancer histology		*	*
Was follow-up long enough for outcome to occur?		*	*	*
**Total Score**		6	7	6

BMI: Body Mass Index

Each star (*) denotes a fulfilled quality assessment criteria

Table S2. Quality assessment of studies using a modified Newcastle-Ottawa scale-CASE-CONTROL STUDY

Quality assessment criteria	Accettable (*)	Campbell et al.
Selection		
Is the case definition adequate?	Pathology-confirmed International Classification of colorectal cancer	*
Representativeness of cases?	Cancer registries/Amsterdam diagnostic criteria	*
Selection of controls?	Same setting as exposed cohort	
Definition of controls?	No evidence of disease	
Comparability		
Stratification for possible modifier of risk	Study stratify for sex	*
Study controls for at least 3 additional	Adjusted for age, smoking habits, alcohol intake, aspirin and/or genes	

Supplementary Table . Quality assessment of studies using a modified Newcastle-Ottawa scale

risk factors?		
Outcome		
Ascertainment of exposure?	Measured BMI	
Same method of ascertainment of cases/controls?		*
Non-response rate?		*
**Total score**		5

Each star (*) denotes a fulfilled quality assessment criteria
